# Cognitive and behavioural but not motor impairment increases brain age in amyotrophic lateral sclerosis

**DOI:** 10.1093/braincomms/fcac239

**Published:** 2022-09-22

**Authors:** Andreas Hermann, Gaël Nils Tarakdjian, Anna Gesine Marie Temp, Elisabeth Kasper, Judith Machts, Jörn Kaufmann, Stefan Vielhaber, Johannes Prudlo, James H Cole, Stefan Teipel, Martin Dyrba

**Affiliations:** Translational Neurodegeneration Section “Albrecht Kossel”, Department of Neurology, University Medical Center Rostock, University of Rostock, 18147 Rostock, Germany; Center for Transdisciplinary Neurosciences Rostock (CTNR), University Medical Center Rostock, University of Rostock, 18147 Rostock, Germany; Deutsches Zentrum für Neurodegenerative Erkrankungen (DZNE) Rostock/Greifswald, 18147 Rostock, Germany; Translational Neurodegeneration Section “Albrecht Kossel”, Department of Neurology, University Medical Center Rostock, University of Rostock, 18147 Rostock, Germany; Deutsches Zentrum für Neurodegenerative Erkrankungen (DZNE) Rostock/Greifswald, 18147 Rostock, Germany; Translational Neurodegeneration Section “Albrecht Kossel”, Department of Neurology, University Medical Center Rostock, University of Rostock, 18147 Rostock, Germany; Deutsches Zentrum für Neurodegenerative Erkrankungen (DZNE) Rostock/Greifswald, 18147 Rostock, Germany; Department of Neurology, University Medical Center Rostock, University of Rostock, 18147 Rostock, Germany; Institute for Cognitive Neurology and Dementia Research, Otto-von-Guericke University Magdeburg, 39120 Magdeburg, Germany; Center for Behavioral Brain Sciences CBBS, 39104 Magdeburg, Germany; Deutsches Zentrum für Neurodegenerative Erkrankungen (DZNE) Magdeburg, 39120 Magdeburg, Germany; Department of Neurology, Otto-von-Guericke University Magdeburg, 39120 Magdeburg, Germany; Deutsches Zentrum für Neurodegenerative Erkrankungen (DZNE) Magdeburg, 39120 Magdeburg, Germany; Department of Neurology, Otto-von-Guericke University Magdeburg, 39120 Magdeburg, Germany; Department of Neurology, University Medical Center Rostock, University of Rostock, 18147 Rostock, Germany; Centre for Medical Image Computing, Department of Computer Science, UCL, London, UK; Dementia Research Centre, Queen Square Institute of Neurology, UCL, London, UK; Deutsches Zentrum für Neurodegenerative Erkrankungen (DZNE) Rostock/Greifswald, 18147 Rostock, Germany; Department of Psychosomatic Medicine, University Medical Center Rostock, University of Rostock, 18147 Rostock, Germany; Deutsches Zentrum für Neurodegenerative Erkrankungen (DZNE) Rostock/Greifswald, 18147 Rostock, Germany

**Keywords:** ageing, frontotemporal dementia, frontotemporal lobar degeneration, motor neurodegenerative diseases, cognitive reserve

## Abstract

Age is the most important single risk factor of sporadic amyotrophic lateral sclerosis. Neuroimaging together with machine-learning algorithms allows estimating individuals’ brain age. Deviations from normal brain-ageing trajectories (so called predicted brain age difference) were reported for a number of neuropsychiatric disorders. While all of them showed increased predicted brain-age difference, there is surprisingly few data yet on it in motor neurodegenerative diseases. In this observational study, we made use of previously trained algorithms of 3377 healthy individuals and derived predicted brain age differences from volumetric MRI scans of 112 amyotrophic lateral sclerosis patients and 70 healthy controls. We correlated predicted brain age difference scores with voxel-based morphometry data and multiple different motoric disease characteristics as well as cognitive/behavioural changes categorized according to Strong and Rascovsky. Against our primary hypothesis, there was no higher predicted brain-age difference in the amyotrophic lateral sclerosis patients as a group. None of the motoric phenotypes/characteristics influenced predicted brain-age difference. However, cognitive/behavioural impairment led to significantly increased predicted brain-age difference, while slowly progressive as well as cognitive/behavioural normal amyotrophic lateral sclerosis patients had even younger brain ages than healthy controls. Of note, the cognitive/behavioural normal amyotrophic lateral sclerosis patients were identified to have increased cerebellar brain volume as potential resilience factor. Younger brain age was associated with longer survival. Our results raise the question whether younger brain age in amyotrophic lateral sclerosis with only motor impairment provides a cerebral reserve against cognitive and/or behavioural impairment and faster disease progression. This new conclusion needs to be tested in subsequent samples. In addition, it will be interesting to test whether a potential effect of cerebral reserve is specific for amyotrophic lateral sclerosis or can also be found in other neurodegenerative diseases with primary motor impairment.

## Teaser

Hermann *et al.* investigated brain ageing in amyotrophic lateral sclerosis. Only amyotrophic lateral sclerosis patients with additional cognitive/behavioural changes showed increased brain ageing while cognitive normal amyotrophic lateral sclerosis patients had even younger brain age than healthy controls, providing a potential cerebral reserve against cognitive/behavioural impairment and faster disease progression.

## Graphical Abstract

**Graphical abstract:**
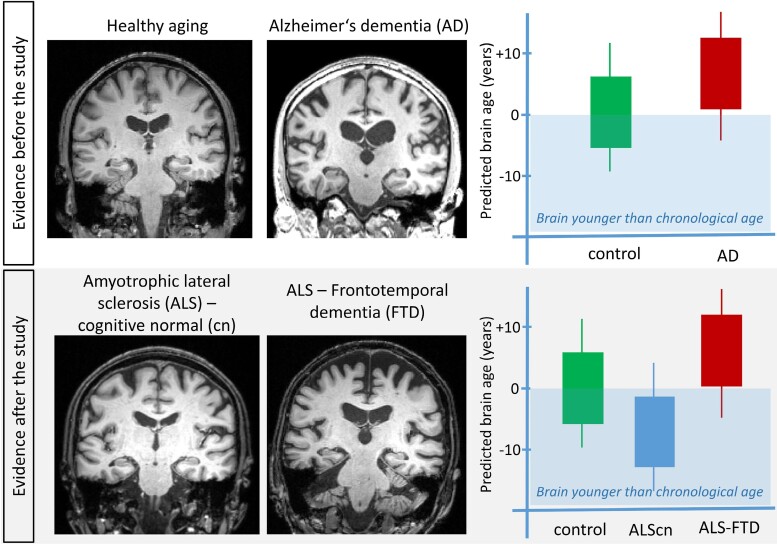

## Introduction

Amyotrophic lateral sclerosis (ALS) is the most common motor neuron disease. It is characterized by upper and lower motor neuron demise, leading to progressive paralysis and death within 1–5 years after symptom onset. On a group level, patients with ALS exhibit central nervous system involvement beyond the upper motor neuron system, including for example the frontotemporal lobes,^1-3^ hypothalamus^4^ and corpus callosum.^3^ Several factors may modify risk and speed of disease progression, including the initial disease manifestation (bulbar versus spinal)^5^ and the extend of frontotemporal impairment.^6^ Cognitive and behavioural impairment accompany motor decline in over half of the patients with ALS over the course of the disease. The most common cognitive deficits in ALS concern executive functions, especially verbal fluency.^7^ The revised consensus criteria of frontotemporal dysfunction in ALS by Strong *et al*.^8^ and by Rascovsky *et al*.^9^ in the case of frontotemporal dementia (FTD) patients with ALS characterize patients according to their cognitive deficits, extent of behavioural impairment and presence or absence of FTD. Notably, cognitive and behavioural impairments predict shorter survival time.^6^

The single most relevant risk factor for sporadic ALS is age, with the highest prevalence of disease in patients over 60 years of age.^10^ This points towards an important role of the ageing process itself. Normal ageing is a process of gradual accumulation of pathologies associated with cognitive and physical decline, which also affects brain volume and nerve cell loss.^11,12^ Thus, ALS might be considered as an increased ageing process of specific brain systems.

Neuroimaging data combined with machine-learning techniques can be used to predict the age of a healthy individual’s brain and allow measuring a potential deviance of an individual’s predicted brain age from chronologic age, termed ‘the predicted brain-age difference’ (PAD).^13^ This approach has been successfully applied not only in both early brain development and ageing in the healthy elderly (for a review see Franke and Gaser^13^), but also in a number of disease conditions, including Alzheimer’s disease, schizophrenia, major depression, multiple sclerosis^14^ and epilepsy (for systematic review see Cole *et al*.^15^ and Wrigglesworth *et al*.^16^). All disease conditions exhibited a remarkably increased PAD score, indicating that brain atrophy exceeded normal brain ageing. This was also true when investigating effects of known cardiovascular risk factors on brain ageing.^17^ In addition, increased PAD scores correlated well with increased mortality or decreased survival in a range of different conditions.^15,18^

To date, there are only few reports on classical ‘motor’ neurodegenerative disorders, such as Parkinson’s disease and none on ALS. Hence, we chose ALS as a paradigmatic motor neurodegenerative disorder, since it has the advantage of clear definitions of motoric and cognitive/behavioural impairment. We hypothesized that patients with ALS would show increased brain age compared with healthy controls (HCs), and that this ageing process would be more pronounced in the presence of additional cognitive and/or behaviour impairment.

## Materials and methods

### Design

This two-centre prospective, observational cross-sectional study was conducted between April 2011 and August 2013. Local ethics committees of both universities approved the study (Rostock: A 2011 56; Magdeburg: 75/11) and all subjects gave written informed consent prior to their inclusion.

General methods used in this study have been already published^19^ and respective details are reported in the Supplementary material. Specific methods relevant to the estimation of brain-age algorithm are described here.

### Participants

We recruited 182 German participants in Rostock and Magdeburg, Germany. Persons with a history of brain injury, epilepsy or psychiatric illness were excluded. Control participants were screened for cognitive impairment using the Montreal cognitive assessment (MoCA) with a cut off of ≤26/30. Seventy HCs and 112 patients diagnosed with ALS according to Swinnen and Robberecht^20^ were included (Supplementary Fig. 1). These cases were characterized into ALS without cognitive/behavioural impairments (ALScn), ALS with cognitive impairment (ALSci), ALS with behaviour impairment (ALSbi), ALS with cognitive and behavioural impairments (ALScbi) and ALS with FTD (ALS-FTD) following the Strong and Rascovsky criteria.^8,9^ Different motoric phenotypes of ALS were classified as classical ALS, upper/lower motor neuron predominant (UMN/LMN) ALS, flail arm, flail leg and progressive muscular atrophy (PMA). None of the patient presented with pure primary lateral sclerosis (PLS). Demographic details are summarized in Table 1.

**Table 1:** Demographic Background of the Participants

	HC (*N* = 70)	ALScn (*N* = 58)	ALSci (*N* = 29)	ALSbi (*N* = 12)	ALScbi (*N* = 5)	ALS-FTD (*N* = 8)	BF_01_ between all ALS & HC
Sex (f/m; %)	40/60	38/62	34/66	33/67	20/80	38/62	4.57
Age at examination	61.00 (10.67)	59.94 (9.74)	62.13 (11.29)	57.40 (12.01)	65.67 (14.62)	61.21 (10.38)	5.88 ± 1.37e − 5%
Education (years)	13.36 (1.62)	13.48 (2.64)	11.86 (1.57)	13.83 (1.99)	11.60 (1.52)	13.00 (2.20)	3.24 ± 8.90e − 6%
MoCA	27.50 (1.29)^a^	25.90 (2.45)	23.6 (3.57)^b^	26.33 (3.42)	21.00 (3.94)^c^	19.13 (5.49)^a^	5.52 ± 8.76e − 6%
Disease duration until TP1 (months)		29.19 (38.76)	30.69 (50.68)^b^	32.00 (30.21)^b^	14.60 (11.55)	12.38 (6.59)	
Total disease duration (months)		63.59 (53.64)	42.81 (33.80)	43.89 (29.02)	30.50 (32.03)	31.75 (18.74)	
Age at onset (years)		57.25 (10.07)	58.35 (12.36)	54.25 (13.23)	63.78 (14.74)	59.44 (10.36)	
EL Escorial criteria at test time (possible/probable/definitive/unknown; %)		38/20/14/28	41/31/14/14	17/42/33/8	20/60/0/20	38/38/25/0	
Onset site (bulbar/spinal/no data; %)		31/51.7/17.2	37.9/44.8/17.2	50/50/0	40/40/20	75/25/0	
ALS-FRSR (as close as possible to test time)	-	39.00 (5.93)	38.07 (6.96)	34.82(4.98)	36.80(5.54)	41.57(3.87)	
Δ ALS-FRSR (as close as possible to test time)		0.65(0.73)	1.40(1.82)^b^	1.12(1.20)	0.91(1.17)	0 .99(0.95)	
Delta ALS-FRSR (at diagnosis)		0.49 (0.40)	0.57 (0.40)	0.89 (0.92)	1.72 (1.67)^a^	0.45 (0.40)	

Matching took place between HC and patients with ALS as a whole. Sex, age and education were matched successfully: independent Student’s *t*-tests supported the absence of differences in age and education, and a χ^2^ test supported the absence of differences in sex distribution. Depicted are mean and SD if not mentioned differentially.

^a^ BF_10_  > 100 in favour of differences to ALSni group.

^b^ BF_10_  > 3 in favour of differences to ALSni group >3.

^c^ BF_10_ > 10 in favour of differences to ALSni group.

### Clinical and neuropsychologic measures

Clinical and neuropsychologic measures were reported previously^19^ and thus reported in the Supplementary material. Examinations were done at the respective recruitment side.

### MRI acquisition and processing

MRI scanning was performed with two 3 T Siemens Magnetom VERIO scanners (Erlangen, Germany) using a 32-channel head coil; one single scanner at each site (Rostock and Magdeburg, Germany). The anatomical T_1_-weighted images were segmented into grey matter, white matter and cerebrospinal fluid partitions using the SPM12 toolbox in Matlab 2019a. Then, the *Diffeomorphic Anatomical Registration Through Exponentiated Lie* (DARTEL) algebra algorithm^21^ was used in combination with a custom brainAgeR brain template to normalize the T_1_-weighted images to the *Montreal Neurological Institute* (MNI) reference coordinate system. The estimated deformation fields were subsequently applied to the grey-matter segments to bring them in MNI space as well, followed by modulation to preserve the total amount of grey matter and smoothing with an 8 mm Gaussian kernel for the voxel-based morphometry (VBM) analysis.

### Brain-age model and PAD

We estimated brain age in R, using the package ‘brainAgeR’, available at https://github.com/james-cole/brainageR. This algorithm was trained on *n* = 3377 healthy individuals and validated on 857 people. To predict brain age, we followed an automated pipeline starting with T_1_-weighted image segmentation and normalization using SPM12 with smoothing with a 4 mm Gaussian kernel to match with the training sample. Then, the spatially normalized grey- and white-matter segments as well as cerebrospinal fluid segments were loaded into R. They were masked to exclude voxels with <30% probability for cerebrospinal fluid, white matter or grey matter, respectively. Subsequently, these segments were vectorized to apply a principal component transformation. The transformed data were then entered in the pretrained Gaussian progress regression model to obtain the predicted brain age. Finally, the predicted age was subtracted from the chronologic age to calculate the PAD. While a positive PAD indicates an older appearing brain, a negative score suggests a younger appearing brain.

### Voxel-based analysis of group differences

Complementarily, we performed a whole-brain VBM analysis for which the normalized and smoothed grey-matter maps were analysed using Statistical Parametric Mapping (SPM12; http://www.fil.ion.ucl.ac.uk/spm). All voxel-based analyses were controlled for total intracranial volume, chronologic age, sex and site, as these were potential nuisance variables. The statistical threshold for the analyses was set to an uncorrected *P* < 0.001 and only clusters with at least 50 voxels extent were retained in the results.

### Statistical analysis

As classical null hypothesis significance testing only enables us to reject the null hypothesis that there are no effects of clinical presentation on PAD, we opted for *Bayes factor hypothesis testing* (BFHT) using an analysis of covariance (ANCOVA). This Bayesian approach allows for the estimation of the likelihood of such effects given the observed data and, hence, more directly infer and compare the actual effects. Specifically, we compared the effects of Strong profile, progressor type, phenotype, onset type, disease duration until MRI scanning and age at disease onset, while controlling for age at MRI, sex and recruitment location by adding them to the null model. We conducted one multi-factorial ANCOVA which compared all these effects against one another, and against the corrected null hypothesis model. A priori, we assumed all models to be equally likely. We applied default Jeffreys-Zellner-Siow priors, with the seed set to 84 293. Please see Table 2 for a summary of the statistical measures we will be reporting. All Bayesian analyses were conducted in *Jeffreys’s Amazing Statistics Program* (JASP, 0.14.3). JASP was set to report the corrected null model on top, and to compare all other models against it using BF_10_. Bayes factors do not require thresholding akin to *P* < 0.05 to determine statistical significance: instead they fall on a continuum ranging from support for the null hypothesis via no support for either hypothesis to support for the alternative hypothesis.^22^ Additionally, we can add qualitative descriptors by stating that BF_10_ > 100 constitutes ‘extreme evidence’ for H_1_, BF_10_ > 30 constitutes ‘very strong’ evidence for H_1_, BF_10_ > 10 constitutes ‘strong’ evidence for H_1_ and BF_10_ > 3 constitutes ‘moderate’ support for H_1_.

**Table 2:** Statistical Measures in Bayesian Probability

Notation/Abbreviation	Full Name	Interpretation
Prior	Prior distribution	Distribution of the effect size, as assumed prior to data collection/analysis
Posterior	Posterior distribution	Actual distribution of the effect size after the data at hand have been analysed
*P*(M)	Prior model probability	Probability of this particular statistical model being supported by the data at hand, as assumed prior to data collection/analysis
*P*(M|data)	Posterior model probability	Posterior probability of this particular model being supported by the data at hand, after they have been analysed
BF	Bayes factor	The strength of evidence in favour of a given statistical model, relative to another statistical model (see below)
BF_01_	Bayes factor 0/1	The strength of evidence in favour of Model 0, relative to Model 1
BF_10_	Bayes factor 1/0	The strength of evidence in favour of Model 1, relative to Model 0
BF_10_ > 100		‘Extreme evidence’ favouring Model 1, relative to Model 0
BF_10_ > 30		‘Very strong evidence’ favouring Model 1, relative to Model 0
BF_10_ > 10		‘Strong evidence’ favouring Model 1, relative to Model 0
BF_10_ > 3		‘Moderate evidence’ favouring Model 1, relative to Model 0
BF_10_ = 1		Model 1 and Model 0 are equally supported by the evidence
BF_10_ < 0.33		‘Moderate evidence’ against Model 1, relative to Model 0 (equivalent to BF_01_ > 3)
BF_10_ < 0.10		‘Strong evidence’ against Model 1, relative to Model 0 (equivalent to BF_01_ > 10)
BF_10_ < 0.03		‘Very strong evidence’ against Model 1, relative to Model 0 (equivalent to BF_01_ > 30)
BF_10_ < 0.01		‘Extreme evidence’ against Model 1, relative to Model 0 (equivalent to BF_01_ > 100)
Error%	Stability of the BF	The range of the BF over the chosen Markov chain Monte Carlo iterations, e.g. BF_10_ = 10 with error% = 20 means that the BF_10_ ranged from 8 to 12
95% CI	Credible interval	With 95% certainty, the true effect size lies within these bounds

### Data availability

**Figure 1:**
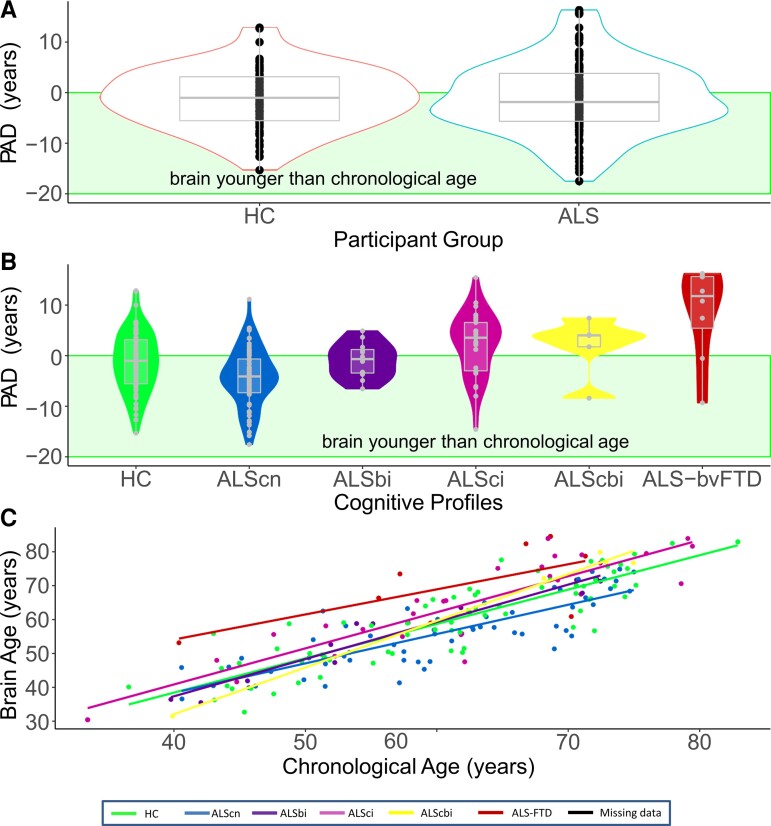
**Predicted brain age difference (PAD) is increased in cognitively/behaviourally impaired patients with ALS.** (**A**) The multivariate model predicted brain age accurately in our healthy controls (HCs). There was no difference in PAD in patients with ALS *per se* (Bayesian independent samples *t*-test, BF_01_ = 5.92, error% = 1.380e − 5, favouring the absence of differences). (**B**) Cognitive/behavioural impairment increased PAD score significantly (ANCOVA main effect, BF_10_ = 524.74), while ALScn patients showed significant decreased PAD (ANCOVA *post hoc* test, BF_10_ = 7.71 in favour of this difference). (**C**) Chronologic age and predicted brain age correlated strongly and had a very narrow credible interval, suggesting a homogeneous, reliable effect (Pearson’s rho for the overall cohort = 0.85, with a 95% credible interval from 0.80 to 0.88; BF_10_ = 2.19e + 48).

**Figure 2:**
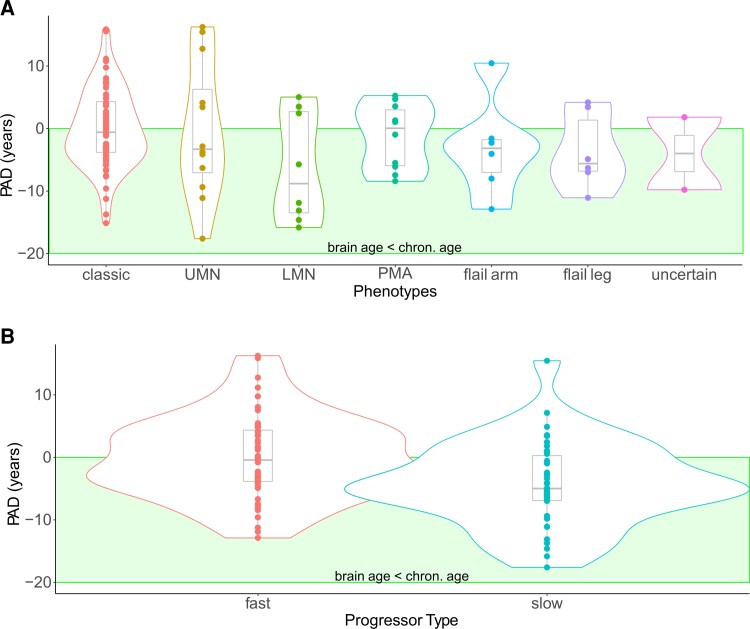
**Predicted brain age is not influenced by motor subtypes but by disease progression rate.** (**A**) Classical motor subtypes did not influence PAD (ANCOVA prior model probability *P*(M) = 2% was reduced to *P*(M|data) < 0.0001% a posteriori). (**B**) The comparison of slow (Δ ALSFRS-R <0.5) versus fast disease progression (Δ ALSFRS-R ≥0.5)—measured by (48-current ALSFRS-R score)/months since disease onset—yielded moderate evidence favouring a main effect (ANCOVA, BF_10_ = 5.52; *post hoc* directional informed ANCOVA BF = 262.61).

**Figure 3:**
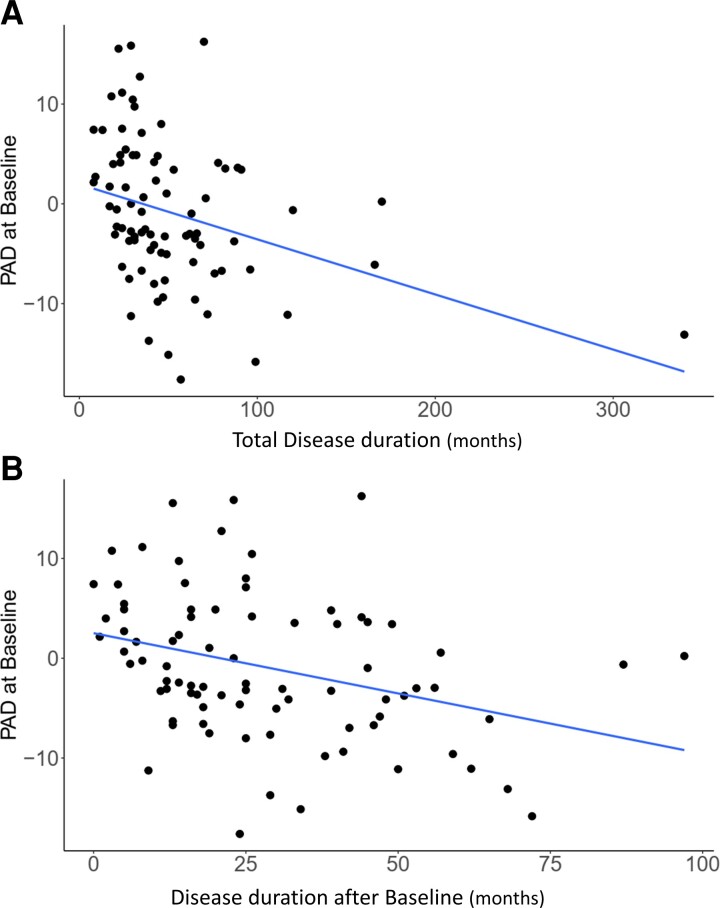
**Predicted brain age is a prognostic marker.** PAD score negatively correlated with total disease duration (**A**, Kendall’s tau = −0.291 with a credible interval from −0.423 to −0.139, BF_10_ = 250.206) and disease duration after baseline (=time point of MRI) (**B**, Kendall’s tau = −0.272 with a 95% credible interval of −0.405 to −0.120, BF_10_ = 96.94**)**.

The original, individual MRI files are not available due to participant confidentiality and privacy concerns. The brainAgeR toolbox is freely available at https://github.com/james-cole/brainageR. The PAD score information was extracted and included in a.csv file, alongside necessary clinical information. These data supporting the BFHT and Figs 1–3 are publicly available from: https://osf.io/fyt7d/, alongside a JASP analysis file, an HTML results file and the R code supporting the figure generation. The MRI data supporting Fig. 4 are not publicly available.

**Figure 4:**
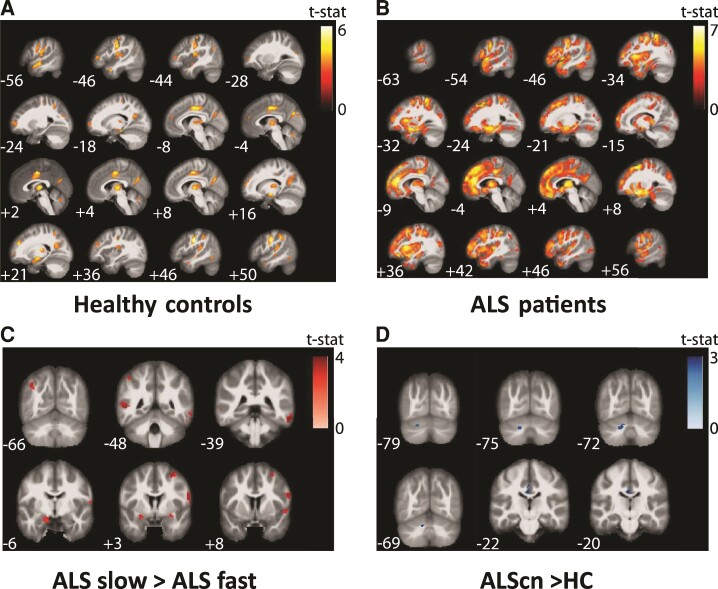
**Correlation of PAD with voxel-based morphometry data showed significantly different patterns between healthy elderly people and patients with ALS.** (**A**) The focal representation of increased PAD score in healthy controls is significant different to the **(B)** disease-associated focal representation of increased PAD of ALS showing a typical frontotemporal atrophy pattern. (**C**) Comparison of voxel-wide grey-matter volumes between ALS fast and slow progressors (ALS slow > ALS fast). (**D**) Comparison of voxel-wide grey-matter volumes between ALScn cases and controls (ALScn > controls). Significant clusters are displayed with T-score values represented by a colour map. An uncorrected threshold of *P* = 0.001 was used for all the presented illustrations and only clusters with at least 50 voxels extent were retained in the results. All clusters shown in **A** and **B** also passed a more conservative significance threshold of *P* = 0.05 applying false discovery rate (FDR) correction. No clusters in **C** and **D** survived FDR correction. All voxel-based analyses were controlled for total intracranial volume, chronologic age, sex and site of measurement as these were potential nuisance variables.

## Results

### Patient cohort

The patients’ onset types included bulbar (*n* = 41), spinal (*n* = 53) or uncertain (*n* = 18). Phenotypically, the patients presented with classical (*n* = 68), upper motor neuron dominant (*n* = 12), flail arm (*n* = 6), flail leg (*n* = 6) or other (*n* = 18) ALS. According to the El Escorial criteria, 40/29/21 patients had a possible/probable/definitive ALS, but 22 patients exhibited pure upper or lower motor neuron involvement and thus did not meet the El Escorial criteria. Patient classification according to the Strong and Rascovsky criteria indicated that most patients were profiled as ALScn (ALS with no cognitive or behavioural impairments, *n* = 58), alongside 29 ALSci, 12 ALSbi, 5 ALScbi and *n* = 8 ALS-FTD patients. All patients underwent genetic testing, with four cases with mutations in *C9ORF72*, three cases of *superoxide dismutase 1* (SOD1), one case of *vesicle-associated membrane protein-associated protein B/C* (VAPB), one case of a juvenile ALS with *senataxin* (SETX) mutation and an uncertain familial link emerging. The remaining patients had sporadic ALS. Demographic and clinical characteristics of the study populations are shown in Table 1. The recruitment flow is shown in Supplementary Fig. 1.

### Predicted brain age was only increased in cognitively/behaviourally impaired patients with ALS

Prior to analysing possible disease effects, we checked that the pretrained brain-age model included in the brainAgeR software was appropriate for our dataset, as it was established on 3377 independent healthy people. For this, we evaluated the PAD (i.e. calculated brain age—chronologic age at time point of MRI) in our HC cohort. As shown in Fig. 1A, the control cohort revealed a (perfectly) matching PAD score of −1.30 ± 6.00 years (mean ± SD) with homogeneous variability across the age range.

We first investigated whether PAD differed between HCs and patients with ALS in general. Surprisingly, patients with ALS in general did not show increased brain age (Fig. 1A). The hypothesis that PAD score of −1.06 ± 7.14 years (mean ± SD) in patients with ALS did not differ from HCs was six times more plausible than the hypothesis that HC and patients would differ (Bayesian independent samples *t*-test, BF_01_ = 5.92, error% = 1.380e − 5). This constitutes moderate evidence that a difference between HC and patients with ALS is absent.

Next, we investigated whether cognitive and/or behavioural impairment influenced brain ageing. Here, we observed moderate to extreme evidence favouring the influence of cognitive/behavioural impairment: the strength of the evidence fluctuated by the severity of impairment. The main effect of Strong profile was 524 times more plausible than the hypothesis that fluctuations of PAD score were driven by age, sex or recruitment location (Table 3, BF_10_ = 524.74, considered ‘extreme evidence’). The ALSci and ALS-FTD patients showed significantly greater brain age compared with ALScn patients and HC (Fig. 1B). ALS-FTD patients’ brains exhibited strong to extreme evidence for greater added ageing (8.58 ± 9.18 years; mean ± SD), compared with the HC (BF_10_ = 221.80, ‘extreme evidence’), ALScn (BF_10_ = 12 918.68, ‘extreme evidence’) and ALSbi groups (BF_10_ = 10.03, strong evidence). ALSci patients’ brains had second highest PAD (2.27 ± 6.40 years, mean ± SD, Fig. 1B); evidence was modest that this was more pronounced than in the HC’s brains (BF_10_ = 4.55), and extremely strong evidence compared with ALScn patients’ brains (BF_10_ = 2735.58). The effects in our data were not strong enough to provide sufficient evidence for or against differences between the ALScbi and ALSbi groups, possibly because these groups exhibited heterogenous effects on PAD as reflected by their large credible intervals including zero (see Supplementary materials). Unexpectedly, we found modest evidence that ALScn patients’ brain age was moderately lower than those of the HC group (−4.33 ± 5.79 years, mean ± SD, BF_10_ = 7.71). In our data, the hypothesis that ALScn patients have younger appearing brains was seven times more likely than the absence of any differences. Predicted brain age correlated well with chronologic brain age in HCs, in ALScn and ALS-impaired (ci, bi, cbi and FTD; Pearson’s rho between 0.66 and 0.99, see Fig. 1C).

**Table 3:** Summary of the Model Comparisons based on the Bayesian ANCOVA

Model Name	*P*(M)	*P*(M|data)	BF_10_	Error%
Null model (incl. sex, age, recruitment location)	0.02	5.02e − 5	1.00	
Strong profile + progressor type	0.02	0.24	4803.70	1.970
Strong profile	0.02	0.03	524.74	3.30
Progressor type	0.02	2.566e − 5	5.52	3.43
Disease duration until examination	0.02	8.55e − 5	1.70	3.21
Phenotype	0.02	2.16e − 5	0.43	3.15
Age at onset	0.02	1.18e − 3	0.37	3.49
Onset type	0.02	3.87e − 4	0.12	3.32
LMN versus UMN	0.02	2.75e − 3	0.77	3.42

BF_10,_ Bayes factor in favour of the model compared with the null model; error%, numerical stability of the BF_10_ over 10 000 MCMC iterations; LMN, lower motor neuron dominant; *P*(M), prior probability of this model; *P*(M|data), posterior probability of this model after data analysis; UMN, upper motor neuron dominant.

The above provides compelling and novel evidence that brain age increases at different speeds across different clinical subgroups of ALS, and that brain age is associated with survival time. We re-ran the above analyses while excluding the 22 patients whose ALS did not meet the El Escorial criteria, and those who had genetic variants of the disease. This did not fundamentally affect the above conclusions with one exception: the difference between ALScn and HC prevailed when either uncertain El Escorial types were excluded (BF_10_ = 7.71; Supplementary Fig. 2), or when genetic variants were excluded (BF_10_ = 7.30; Supplementary Fig. 3). Therefore, we did not exclude those for further analysis, being most likely more representative for typical clinical settings.

### Predicted brain age was not influenced by motor subtypes but by disease progression rate

Different motoric phenotypes of ALS—classical ALS, UMN/LMN ALS, flail arm, flail leg, PMA—did not exhibit differences in brain ageing (Fig. 2A): motoric phenotype effect decreased in plausibility from the prior model probability *P*(M) = 2% to a posterior model probability *P*(M|data) < 0.0001%. Consequently, the Bayesian analysis of effects demonstrated that models excluding the motoric phenotype variable were four times better than those including phenotype (BF_excl_ = 4.60), and models excluding disease onset site were five times better than those including disease onset site (BF_excl_ = 4.94). The plausibility of onset site’s effect on brain ageing also decreased from 1.6% to below 0.0001% (Supplementary Fig. 4A). In summary, clinicomotoric aspects of ALS did not affect brain ageing. Our data further supported the absence of correlations between increased brain ageing and age at disease onset (Pearson’s *r* = 0.03 with a 95% credible interval ranging from −0.155 to 0.212, BF_01_ = 8.06), and disease duration until the time point of MRI investigation (Pearson’s *r* = −0.127 with a 95% credible interval between −0.301 and 0.060, BF_01_ = 3.54; Supplementary Fig. 5).

We next asked whether upper motor neuron involvement was the key driver of increased PAD score, so we grouped the PMA and LMN groups (including flail-arm and flail-leg syndrome) and compared them with all others. However, the evidence regarding a potential effect of upper motor neuron involvement was inconclusive: our data decreased the effect’s plausibility by a factor of 10^4^ but at BF_10_ = 0.77, no hypothesis was preferable to the other (Supplementary Fig. 4B).

Several clinical studies reported differential therapeutic effects in rapid versus slow-progressing patients with ALS suggesting that rapid disease progression might represent a distinct disease type. Thus, we directionally hypothesized that fast progressors—dichotomized by a monthly decline of ALS functional rating scale revised (ALSFRS-R) ≥0.5—would exhibit increased brain age. There was moderate evidence favouring the main effect of Progressor type (Table 3, BF_10_ = 5.52) which we followed up with an informed Bayesian ANOVA to specifically test our directional hypothesis. It was 262.61 times more plausible than the effects of chronologic age, sex and recruitment location alone. As shown in Fig. 2B, slow progressors’ brains were younger than their chronologic age (−4.25 ± 6.75 years). Of note, lower PAD in slow progressors was independent of their Strong profiles (Supplementary Fig. 4C).

The hypothesis that slow-progressing ALScn patients had younger brains than HC was 62 times more plausible than the absence of an effect (BF_+0_ = 62.45, Supplentary Fig. 4D). This suggests that these patients might possess the strongest resilience factors protecting them from cognitive/behavioural impairment or from brain atrophy/ageing.

### Cognitive/behavioural impairment and disease progression were independent but additive predictors of PAD

A posteriori, the most plausible effects in our data were the co-occurring but independent main effects of Strong profile and progressor type: they increased in probability from *P*(M) = 1.6% to *P*(M|data) = 24% and were nearly 5000 more likely than the sole influences of chronologic age, sex and recruitment location (*P*(M) = 0.016, *P*(M|data) = 0.240, BF_10_ = 4803.70, error% = 3.57). This ANCOVA was also able to discriminate between suitable and unsuitable predictors. Models containing Strong profile were 579 times better than those without this predictor (*P*(incl) = 0.500, *P*(incl|data) = 0.998, BF_incl_ = 579.20), and models containing progressor type were seven times better than models without it (*P*(incl) = 0.500, *P*(incl|data) = 0.884, BF_incl_ = 7.31) when it came to explaining PAD scores. This informs us that Strong profile was the more plausible predictor for brain ageing but both were independently relevant (Table 3).

### PAD correlated with survival time

We next investigated predictive power of brain ageing on disease duration/survival. Survival data were available for 83 patients. Firstly, the correlation between increased PAD and shorter total disease duration was 250 times more plausible than the absence of any correlation (Kendall’s tau = −0.291 with a credible interval from −0.423 to −0.139, BF_10_ = 250.206; Fig. 3A). The total disease duration was estimated based on patients’ memory of their own disease onset. In addition, we correlated the disease duration from time point of MRI to death and PAD. The correlation between older appearing brain and shorter survival was 97 times more plausible than the absence of any correlation (Kendall’s tau = −0.272 with a 95% credible interval of −0.405 to −0.120, BF_10_ = 96.94; Fig. 3B).

### What is the focal representation of increased brain age in ALS?

We were wondering to what degree PAD score correlated with motor cortex atrophy. In addition, we tested with which brain volumes PAD score was associated in controls. Correlation of PAD and whole-brain grey-matter maps showed significantly different patterns between healthy elderly people and patients with ALS. While in healthy elderly people, the focal representation of increased PAD score was mainly seen in midcingulate cortex, rolandic operculum and postcentral gyrus (Voxels >1000; *t*-score >4.5; uncorrected *P* < 0.001; Fig. 4A), patients with ALS showed remarkable focal atrophy in frontotemporal and motor cortex as well as in the thalamus (Fig. 4B). Thus, PAD was associated with motor cortex atrophy in both, HCs and patients with ALS. This also means that motor cortex atrophy was not correlated with PAD score exclusively in patients with ALS (Supplementary Fig. 6A and B). Frontobasal structures distinguished the PAD-VBM correlations between ALScn compared with ALSi (ci, bi, cbi and FTD; Supplementary Fig. 6C) or HC compared with ALSi (Supplementary Fig. 6D).

### What are possible resilience factors?

We next investigated which focal brain map patterns contributed best to the younger brain age in slowly progressive patients with ALS. We compared voxel-wide grey-matter volumes between ALS fast versus slow progressors and identified significant regional atrophy mainly in the operculum and temporal lobe (Voxels ∼1000, *t*-value >4.0; Fig. 4C) in fast compared with slow progressors.

We finally studied the surprisingly younger brain age in ALScn patients and compared voxel-wide grey-matter volumes between ALScn cases and controls. We identified few and only very small significant focal atrophy patterns (Voxels <50, *t*-value >3.5) in ALScn patients compared with HCs. Of note, however, we detected relative increase of grey-matter volume in ALScn patients compared with controls in left Crus II and left Lobule VIIa (Voxels >250, *t*-values >3.0; Fig. 4D).

## Discussion

We used volumetric MRI with data-driven machine-learning algorithms to estimate individuals’ brain age in patients with ALS and age-matched controls. We had hypothesized that PAD would be increased in motor impairment-only ALS cases and that this effect would even be more pronounced in the presence of additional cognitive and behaviour impairment. PAD was a very stable parameter of the individual, neither affected by age of onset, motor subtypes, disease onset type or disease duration until time point of investigation. Against our a priori hypothesis, we found strong evidence that predicted brain age was not increased in ALS *per se*; however, higher PAD was observed in patients with ALS who were additionally cognitively and/or behaviourally impaired. Surprisingly, predicted brain age was lower in ALScn patients and the subgroup of slowly progressive patients with ALS when compared with HCs. While a significant number of studies reported increased PAD in disease conditions such as Alzheimer’s disease, multiple sclerosis, epilepsy or schizophrenia, none have reported reduced/younger brain ages in a disease condition.

To better understand the unexpected results of younger brain age in ALScn compared with controls, we went on to investigate correlations between PAD and grey-matter volume in a VBM analysis. Our initial hypothesis was that the correlate for PAD score variance in ALS was motor cortex atrophy. Indeed, PAD correlated with motor cortex grey matter, but also with large areas outside of motor cortex (Fig. 4 and Supplementary Fig. 6). PAD score was associated with frontotemporal lobe atrophy, consistent with pattern of brain atrophy found in ALS cases with cognitive and/or behavioural impairment. Furthermore, focal temporal lobe atrophy pattern was the morphologic correlate of increased PAD score in fast versus slow progressors (Fig. 4C). Similar atrophy patterns were recently reported in other VBM studies.^23^ Together these data inform us that the used machine-learning algorithm was sensitive enough to detect changes typically found in patients with ALS and distinct ALS populations.

One of the key findings was the surprisingly lower brain age of ALScn patients compared with HCs and the increased relative brain volume in cerebellar structures. Of note, Qiu and colleagues^24^ reported increased grey-matter volume in cerebellar subregions in a VBM study. Similarly, Zhou and colleagues^25^ reported an increased brain functional network connectivity in the cerebellum of patients with ALS. Since both studies excluded patients with an FTD diagnosis and cognitive impairment (MoCA <26), these patients very much resembled our ALScn group. The cerebellum contributes to executive functions such as planning, verbal fluency, abstract reasoning and working memory.^26^ All of these functions are typically impaired in ALSi patients. Thus, cerebellar compensation may be specifically important in cognitively unimpaired patients with ALS and thus could be considered as resilience factor against executive dysfunction associated with shortened survival.

Consequently, the question arises as to how this translates, e.g. to C9ORF72-ALS patients, the most common monogenetic form of ALS. In C9ORF72 patients, most abundant dipeptide repeat–associated neuropathology is found in the cerebellum.^27^ It is remarkable though that C9ORF72 patients do much more often suffer from cognitive/behavioural impairment and shorter survival than sporadic patients with ALS.^28^ In agreement with these data, our findings suggest a cerebellar resilience factor against cognitive/behavioural impairment associated with longer survival in patients with ALS which needs further testing in independent cohorts.

We cannot yet generalize whether motor system neurodegeneration without cognitive affection does not lead to accelerated brain ageing also in other motor neurodegenerative diseases such as Parkinson’s disease, or whether this is a specific finding for ALS. Only two studies on Parkinson’s disease have been published so far, both reporting ‘surprisingly’ small increases in PAD,^29^ especially when compared with Alzheimer’s disease.^30^ Of note, neither study distinguished systematically between demented, cognitively impaired and non-demented Parkinson’s disease patients. However, they showed a negative correlation between cognitive performance (measured by MoCA) and PAD. Thus, future studies are needed, separating Parkinson’s disease patients with and without (mild) cognitive impairment to address this question.

Limitations of the study are the small sample sizes of ALS subgroups, specifically in the ALSbi, −cbi and –FTD subgroups, which is also true for the motoric phenotypes. This did not allow us to analyse all suspected confounding factors such as diets, environmental pollutants, trauma, drug use, etc. Thus, there is considerable heterogeneity in the whole cohort which might explain some of the variances, e.g. in disease duration. However, this distribution reflects the population incidence of motor subtypes and cognitive/behavioural impairment, as ALS presents very heterogeneously.^20^ Importantly, small sample sizes serve to detect large or very large effects, as reported here. However, this limitation does not apply to the overall results of a negative PAD in ALScn and positive PAD only in case of additional cognitive and/or behavioural impairment. Nevertheless, larger follow-up studies are warranted to further determine PAD and the underlying processes in the different forms of ALS. Furthermore, the brain-age analysis pipeline yields a single value and its ease of use might make it well suited in routine clinical care. However, it is conceptualized on the whole brain and distinct neuroanatomical information are not available. Consequently, it might not be sensitive enough for every disease entity depending on the spatial patterns of brain atrophy.

Therefore, we performed extensive correlations using VBM analysis. By doing so, we can conclude that we showed here the value of brain-age algorithms in the motor neurodegenerative disease ALS. In addition, we report unexpected findings of younger brain age in patients with ALS without cognitive/behavioural impairments (‘cognitive normal’) not only if compared with ALSi (=ci, bi, cbi or FTD) patients but even if compared with HCs with possible cerebellar resilience factors against cognitive/behavioural impairment in ALS.

## Supplementary Material

fcac239_Supplementary_Data

## Abbreviations

ALS =: amyotrophic lateral sclerosis
ALSFRS-R =: ALS functional rating scale revised
ALScn =: ALS without cognitive/behavioural impairments (‘cognitive normal’)
ALSci =: ALS with cognitive impairment
ALSbi =: ALS with behavioural impairment
ALScbi =: ALS with cognitive and behavioural impairments
ALS-FTD =: ALS with frontotemporal dementia
HCs =: healthy controls
LMN ALS =: lower motor neuron predominant ALS
MoCA =: Montreal cognitive assessment
PAD =: predicted brain age difference
PMA =: progressive muscular atrophy
UMN ALS =: upper motor neuron predominant ALS
VBM =: voxel-based morphometry
