# Supplementary material for: Cognitive and behavioural but not motor impairment increases brain age in amyotrophic lateral sclerosis
Source: Brain Commun. 2022 Sep 22;4(5):fcac239. doi: 10.1093/braincomms/fcac239 (PMC9556938; doi:10.1093/braincomms/fcac239)
Supplement: fcac239_Supplementary_Data [file fcac239_supplementary_data.docx]

**Supplement Methodology (Full methods)**

**Design**

This was a two-center prospective, observational cross-sectional and longitudinal study conducted between April 2011 and August 2013. The local ethics committees of both universities approved the study (Rostock: A 2011 56; Magdeburg: 75/11) and all subjects gave written informed consent prior to their inclusion.

**Participants**

We recruited 182 German participants in Rostock and Magdeburg, Germany. Persons with a history of brain injury, epilepsy or psychiatric illness were excluded. Control participants were screened for cognitive impairment using the Montreal Cognitive Assessment, and excluded if they scored below 26 out of total 30. 70 healthy controls and 112 patients diagnosed with ALS using the revised El Escorial criteria were included ^1^ (Supplemental Fig. 1). ALS cases were classified into ALS without cognitive/behavior impairments (ALScn), ALS with cognitive impairment (ALSci), ALS with behavior impairment (ALSbi), ALS with cognitive and behavioural impairments (ALScbi) and ALS with frontotemporal dementia (ALS-FTD) following the Strong and Rascovsky criteria^2,3^. Demographic details can be found in Table 1

**Clinical and neuropsychological measures**

*Cognitive testing*. Participants underwent full neuropsychological examination in the executive, memory, visuospatial and fluency domains, for details see Kasper, Schuster, Machts, Bittner, Vielhaber, Benecke, Teipel and Prudlo ^4^. Where necessary, we corrected cognitive tests for motor impairment ^5^. We calculated the patients’ standardized z-scores based on the controls’ means and standard deviations; z scores ≤ -2 were considered as impaired performance.

*Behavioural assessment*. For behavioural classification, we relied on clinical observations and proxy-rated *Frontal Systems Behavior Scales* ^6^. Ratings were transformed to T-scores based on published norms, with T≥65 reflecting behavioural impairment.

*Motor impairment.* We used the revised ALS-Functional Rating Scale ALSFRS-R, ^7^, and calculated patients’ progression rate δ as: (48-current ALSFRS-R score)/months since disease onset. Participants with δ≥0.5 were classified as “fast progressors” (n=56). Participants with δ<0.5 were considered “slow progressors” (n=38).

**MRI acquisition and processing**

MRI scanning was performed with two 3T Siemens Magnetom VERIO scanners (Erlangen, Germany) using a 32-channel head coil; one single scanner at each site (Rostock and Magdeburg, Germany). High-resolution T_1_-weighted anatomical images were acquired using the magnetization-prepared rapid gradient echo (MPRAGE) sequence with the following parameters: 256x256 image matrix with 192 sagittal slices, FOV 250x250x192mm, voxel size 1x1x1mm³, echo time 4.82ms, repetition time 2500ms, and flip angle 7°. The anatomical T_1_-weighted images were segmented into grey matter, white matter and cerebrospinal fluid partitions using the SPM12 toolbox in Matlab 2019a. Then, the *Diffeomorphic Anatomical Registration Through Exponentiated Lie (DARTEL)* algebra algorithm ^8^ was used in combination with a custom brainAgeR brain template to normalize the T_1_-weighted images to the *Montreal Neurological Institute (MNI)* reference coordinate system. The estimated deformation fields were subsequently applied to the grey matter segments to bring them in MNI space as well, followed by modulation to preserve the total amount of grey matter and smoothing with an 8 mm Gaussian kernel for the voxel-based morphometry analysis. In phantom tests according to the American College of Radiology guidelines ^9^, both sites’ scanners met the criteria for geometric accuracy, high contrast spatial resolution, slice thickness accuracy, slice position accuracy, image intensity uniformity, percent signal ghosting and low contrast object detectability.

**Brain Age model and predicted Brain Age Difference (PAD)**

For Brain Age estimation, we used the brainageR toolbox version 2.1 available at https://github.com/james-cole/brainageR. This model is implemented in R (https://www.r-project.org/), and had been trained on n= 3377 healthy individuals and validated on 857 people. For the prediction of Brain Age, it follows an automated pipeline starting with T1-weighted image segmentation and normalized using SPM12 and smoothing with an 4 mm Gaussian kernel. Then, the spatially normalized grey and white matter segments as well as cerebrospinal fluid segments were loaded into R, masked to exclude voxels with less than 30% tissue/fluid probability, and vectorized to apply a principal component transform. The transformed data was then entered in the pretrained Gaussian progress regression model to obtain the predicted brain age. Finally, the predicted age was subtracted from the chronological age to calculate the “predicted brain age difference” (PAD):

***Predicted age difference (PAD) = Estimated Brain Age – Chronological Brain Age***

While a positive PAD indicates an older appearing brain, a negative score suggests a younger appearing brain.

**Voxel-based analysis of group differences**

Complementarily, we performed a whole brain voxel-based morphometry analysis for which the normalized and smoothed grey matter maps were analyzed using Statistical Parametric Mapping (SPM12; http://www.fil.ion.ucl.ac.uk/spm).

First, we run a linear regression model correlating the PAD scores with gray matter volume to determine potential regional pattern of atrophy driving the PAD score. We conducted this analysis separately for the healthy controls and the ALS patients.

Furthermore, we performed group comparisons to investigate the brain volume differences between different clinical subgroups. On one side, we investigated the between group differences between the healthy controls, the ALS non-impaired (ni) and the ALS impaired groups through a full factorial model design. Here, ALS impaired group included the Strong subgroups bi, ci, cbi, and bv-FTD. Notably, the contrast vector was weighted by the size of these cognitive subgroups such that each patient was equally weighted. On the other side, a two-sample T-test design was run between ALS fast and slow progressors, dichotomized by a monthly decline of ALSFRS-R ≥0.5.

All voxel-based analyses were controlled for total intracranial volume (TIV), chronological age, sex and site as these were potential nuisance variables. The statistical threshold for the analyses was set to an uncorrected p<0.001 and only clusters with at least 50 voxels extent were retained in the results.

**Statistical Analysis**

As classical null hypothesis significance testing only enables us to reject the null hypothesis that there are no effects of clinical presentation on PAD, we opted for *Bayes factor hypothesis testing (BFHT)* using an analysis of covariance. This Bayesian approach allows for the estimation of the likelihood of such effects given the observed data and, hence, more directly infer and compare the actual effects. Specifically, we compared the effects of Strong profile, progressor type, phenotype, onset type, disease duration until MRI scanning, and age at disease onset, while controlling for age at MRI, sex and recruitment location by adding them to the null model. We conducted one multi-factorial analysis of covariance (ANCOVA) which compared all these effects against one another, and against the corrected null hypothesis model. A priori, we assumed all models to be equally likely.

We applied default Jeffreys-Zellner-Siow (JZS) priors, with the seed set to 84293. Please see Table 2 for a summary of the statistical measures we will be reporting. All Bayesian analyses were conducted in *Jeffreys’s Amazing Statistics Program* (JASP, 0.14.3). JASP was set to report the corrected null model on top, and to compare all other models against it using BF_10_. Bayes factors do not require thresholding akin to p<.05 to determine statistical significance: instead they fall on a continuum ranging from support for the null hypothesis via no support for either hypothesis to support for the alternative hypothesis^10^. Additionally, we can add qualitative descriptors by stating that BF_10_>100 constitutes “extreme evidence” for H_1_, BF_10_>30 constitutes “very strong” evidence for H_1_, BF_10_>10 constitutes “strong” evidence for H_1_ and BF_10_>3 constitutes “moderate” support for H_1_.

**Supplementary Figures**

**
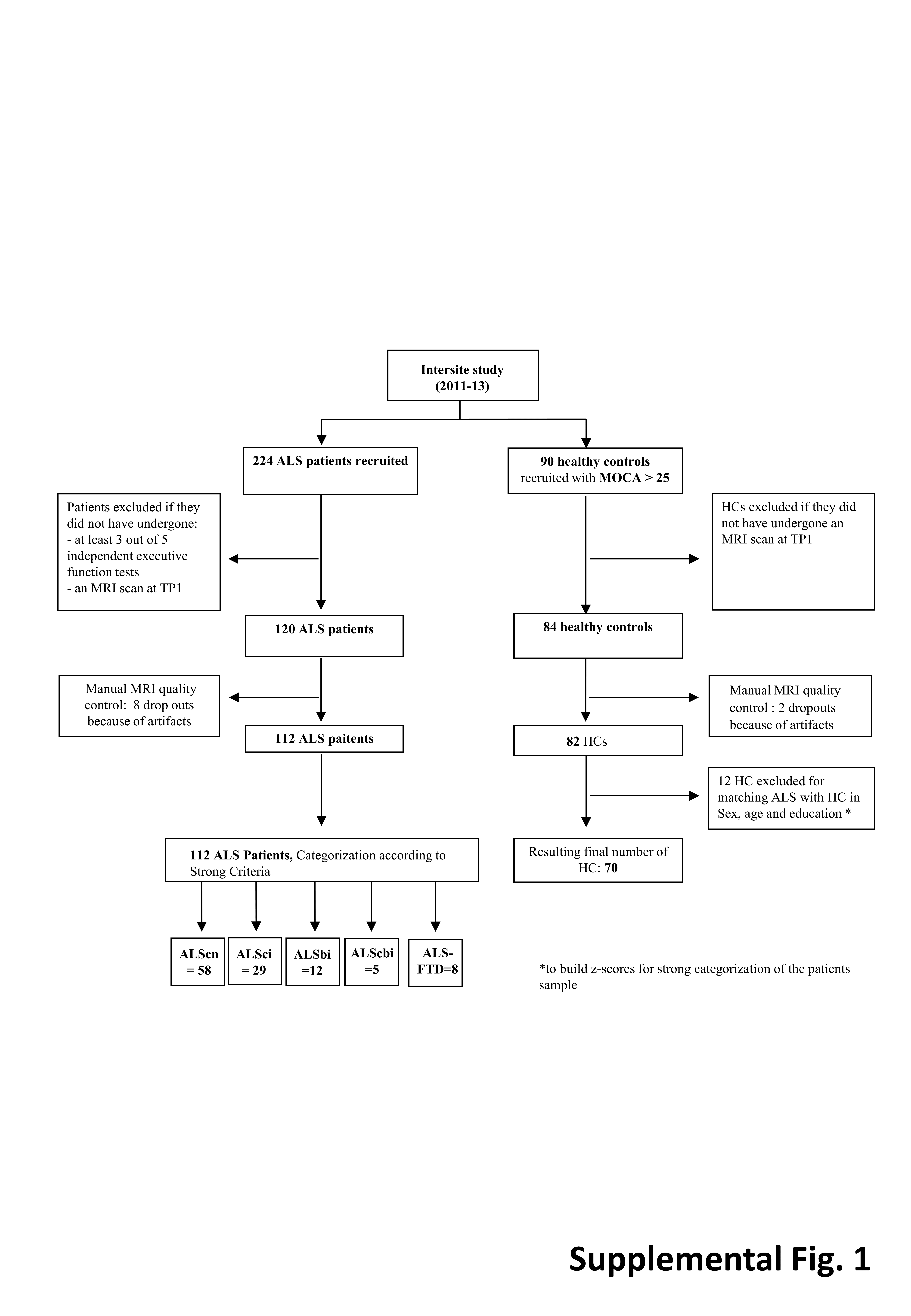
**

**Supplementary Figure 1:** Flow chart of the study population.


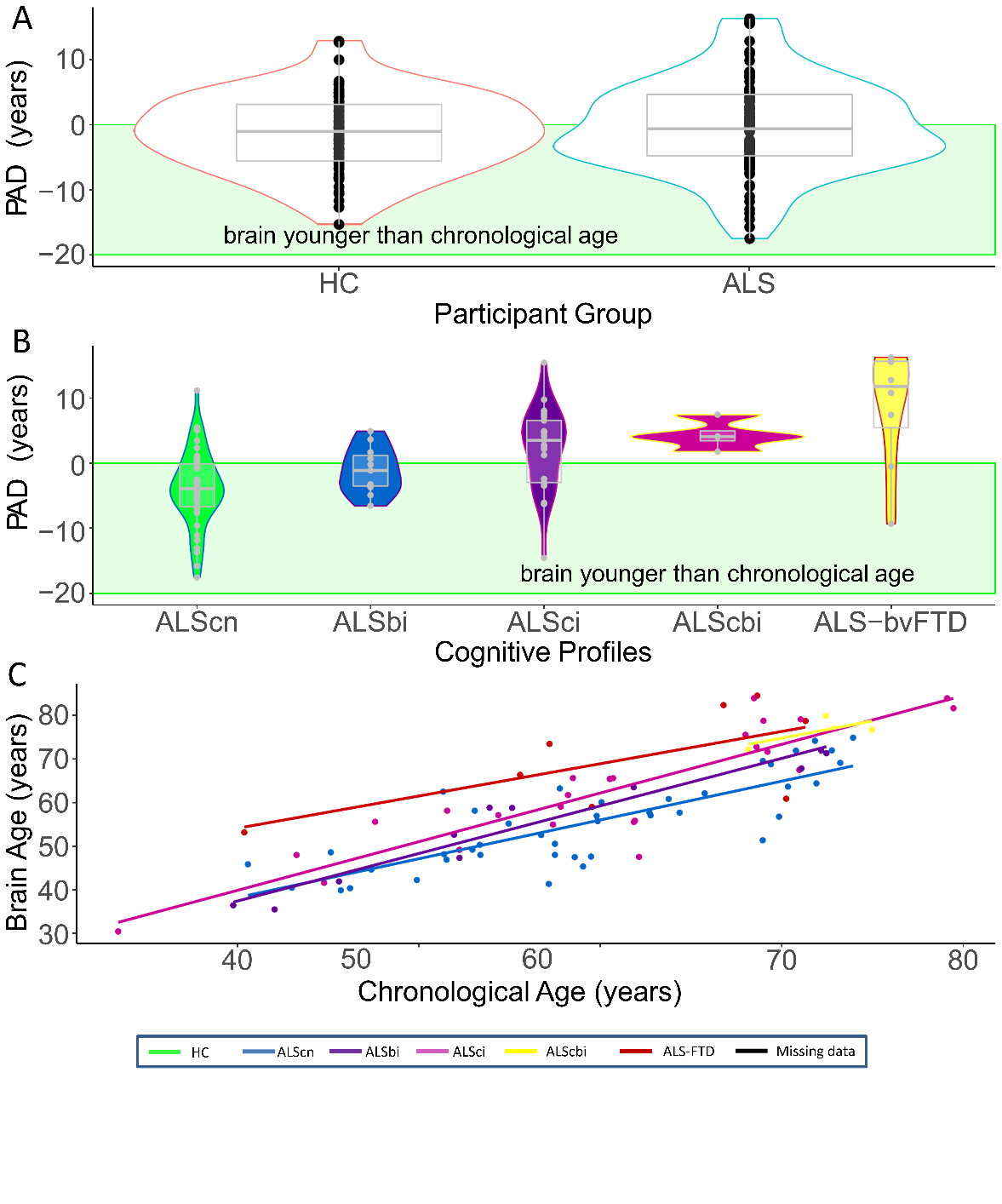


**Supplementary Figure 2: Predicted brain age difference (PAD) is increased in cognitively/behaviorally impaired ALS patients also if patients without meeting El Escorial criteria are excluded.** **(A)** There was no difference in PAD in ALS patients per se. **(B)** Cognitive/behavioral impairment increased PAD score significantly, while the difference between ALScn and HC prevailed when uncertain El Escorial types were excluded (BF_10_=7.71). **(C)** Chronological age and predicted brain age correlated strongly and had a very narrow credible interval, suggesting a homogeneous, reliable effect.

**
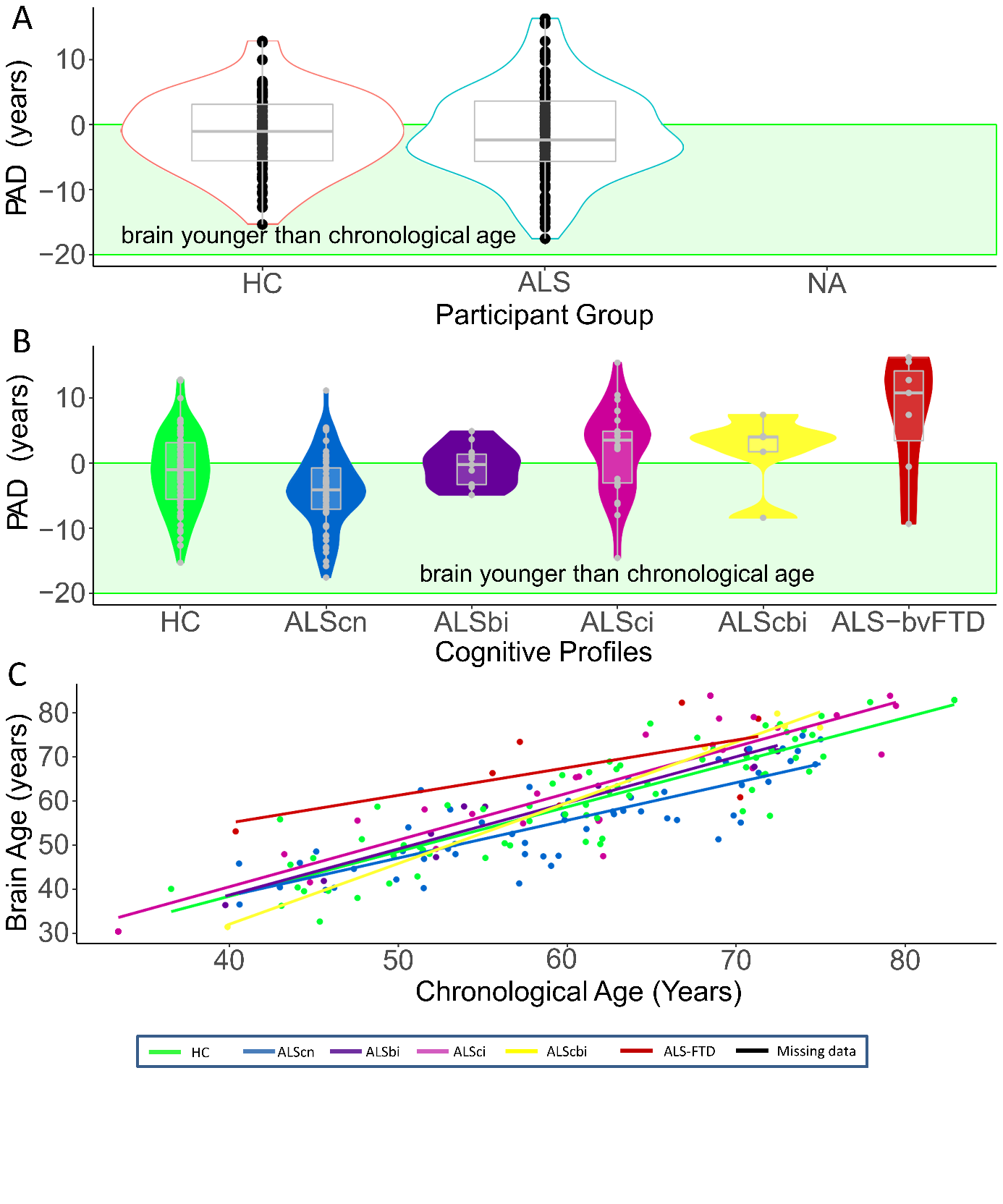
**

**Supplementary Figure 3: Predicted brain age difference (PAD) is increased in cognitively/behaviorally impaired ALS patients also if patients with monogenetic ALS forms were excluded.** **(A)** There was no difference in PAD in ALS patients per se. **(B)** Cognitive/behavioral impairment increased PAD score significantly, while the difference between ALScn and HC prevailed when genetic variants were excluded (BF_10_=7.30). **(C)** Chronological age and predicted brain age correlated strongly and had a very narrow credible interval, suggesting a homogeneous, reliable effect.


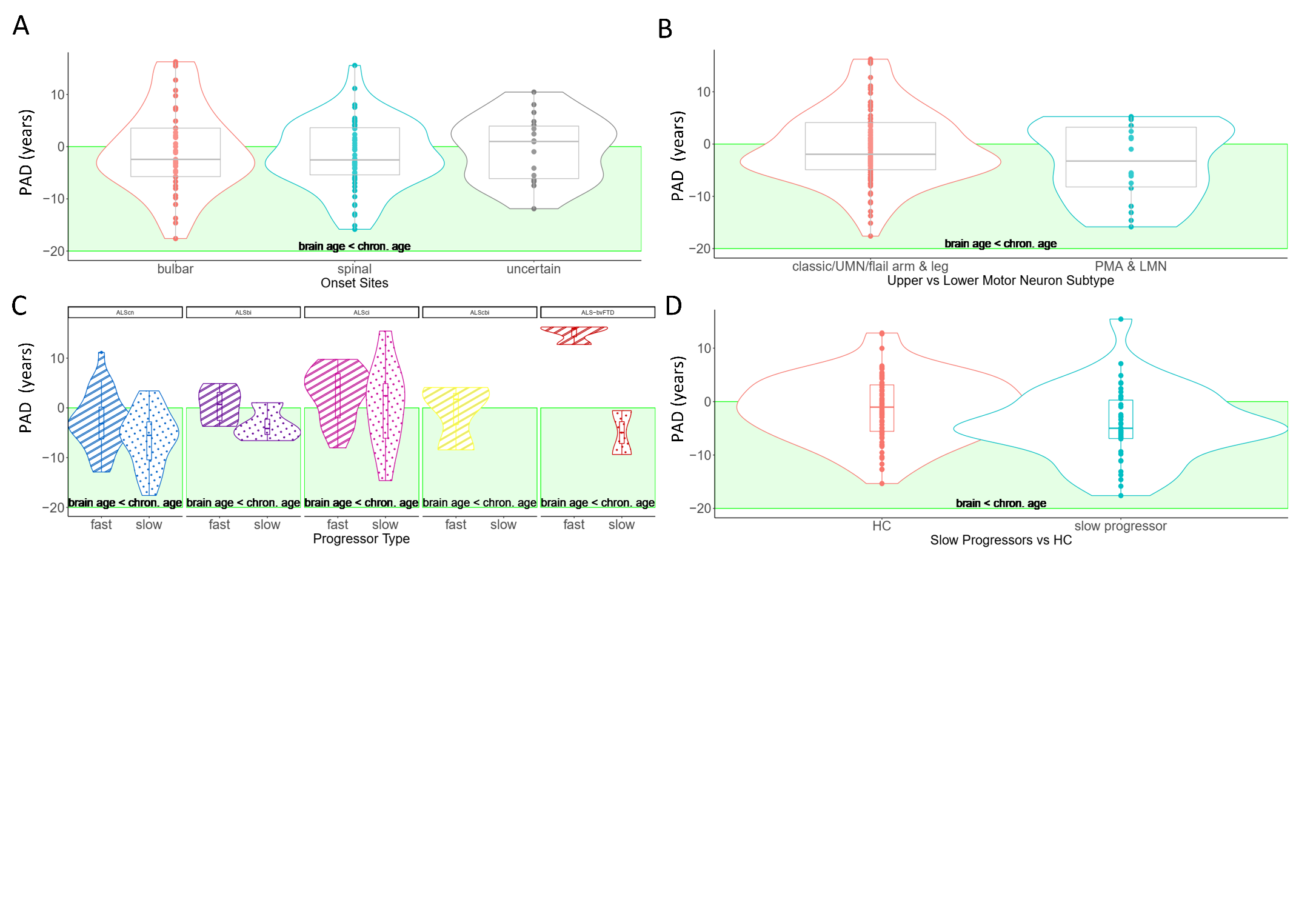


**Supplementary Figure 4: Predicted brain age is not influenced by motor subtypes but by disease progression rate. (A)** Site of disease onset did not influence PAD (with its prior probability in the ANCOVA decreasing from 1.6% to below 0.0001%). **(B)** Upper motor neuron involvement was also not probable as a predictor of PAD: our data decreased the effect’s plausibility by a factor of 10^4^. **(C)** The comparison of slow (Δ ALSFRS-R <0.5) *vs.* fast disease progression (Δ ALSFRS-R ≥0.5) – measured by (48-current ALSFRS-R score)/months since disease onset – yielded moderate evidence favouring a main effect in every subgroup of the Strong criteria (ANCOVA combined main effects BF_10_=4803.70). **(D)** Slowly progressive ALS patients had younger predicted brain age than healthy controls (directional Bayesian independent samples t-test, BF_+0_=62.45).


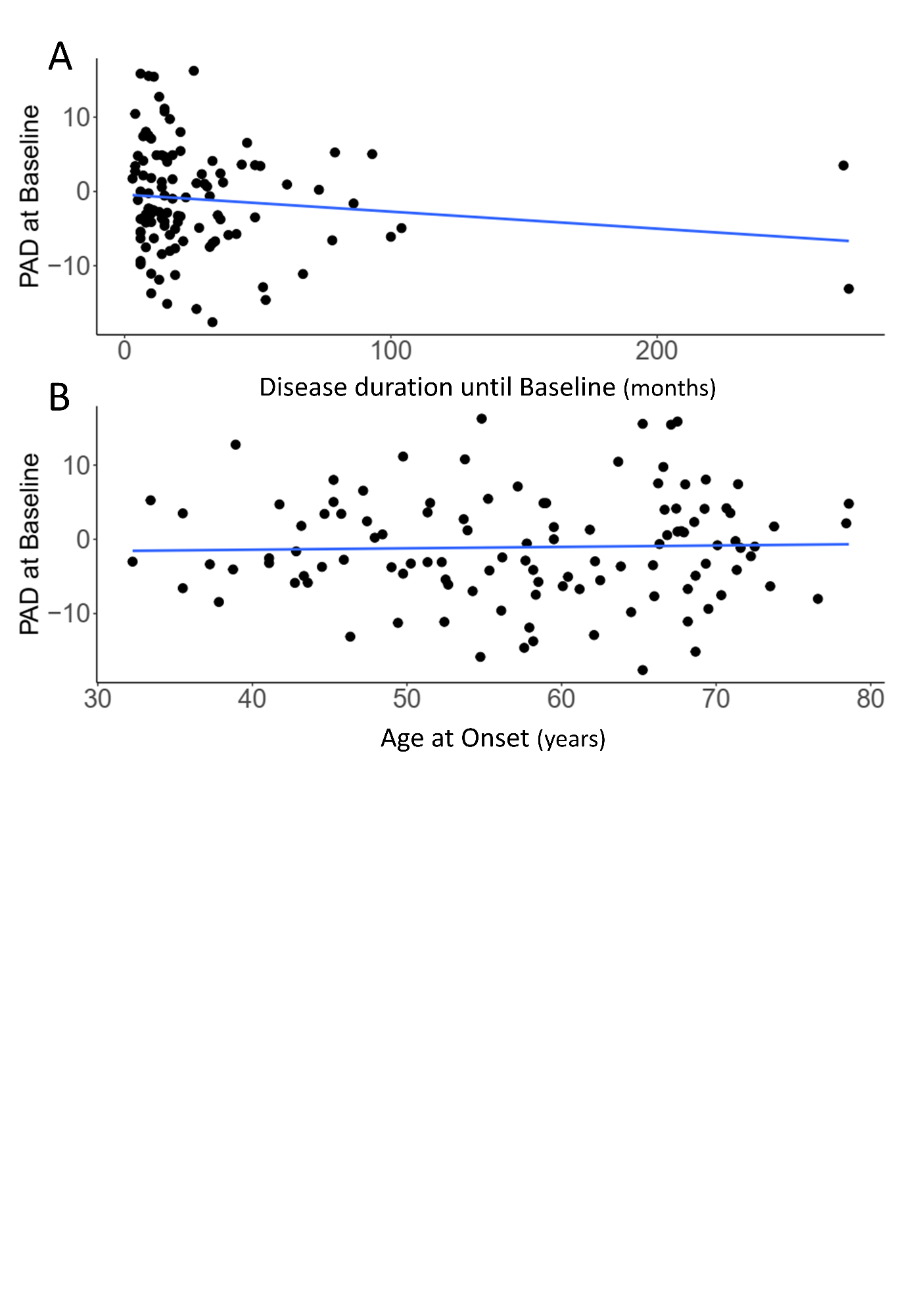


**Supplementary Figure 5: PAD score is a stable parameter.** PAD did neither correlate with disease duration until timepoint of the MRI measurement (=baseline) **(A)** nor with age at onset **(B)**.


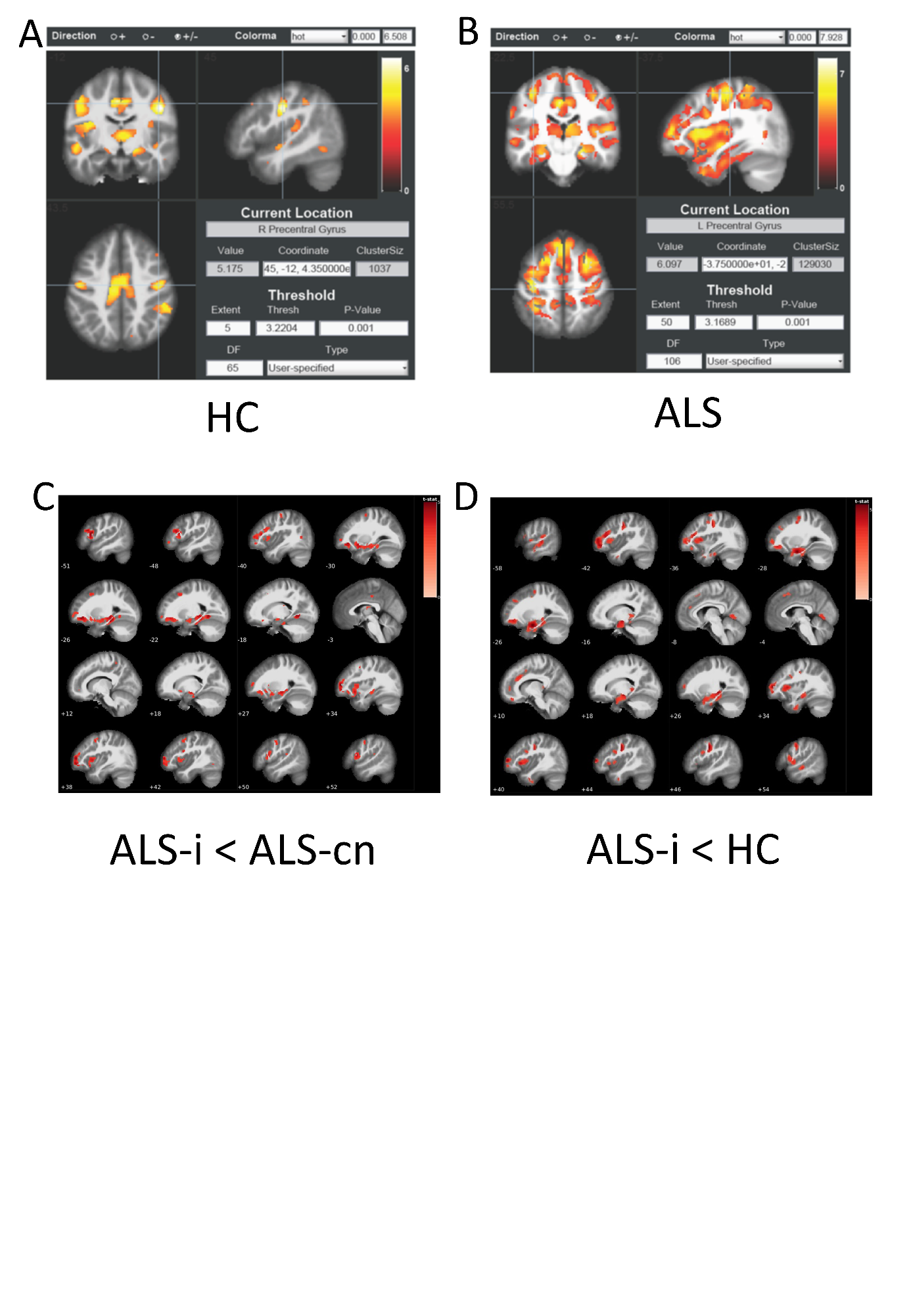


**Supplementary Figure 6: Correlation of PAD with brain maps showed that motor cortex only partially caused the increased PAD score in ALS.** **(A-B)** Significant clusters are displayed with color map representing T-score values on slices in axial, coronal and sagittal orientation, focusing on the precentral gyrus. Age-associated atrophy pattern in healthy elderly people **(A)** and disease-associated atrophy pattern in ALS patients **(B)** shows involvement of motor cortex and non-motoric regions in both. Same tresholds are used as for Figure 4. Represented current location (cross of lines) represents R/L precentral gyrus, respectively. Note the different maximum T-value and cluster size of the precentral gyrus in ALS and Controls. Disease-associated atrophy pattern in ALS showed larger effect (cluster size) in motor cortex. Furthermore, in ALS patients, significant more regions contributing to the PAD deviance, mainly in frontotemporal structures. **(C-D)** These frontobasal structures contributed to PAD deviance in ALSi patients (ALS impaired < cognitively normal **(C)** and ALS impaired < healthy controls **(D)**)**.**

**References**

1. Brooks BR, Miller RG, Swash M, Munsat TL, World Federation of Neurology Research Group on Motor Neuron Diseases. El Escorial revisited: revised criteria for the diagnosis of amyotrophic lateral sclerosis. *Amyotroph Lateral Scler Other Motor Neuron Disord*. Dec 2000;1(5):293-9. doi:10.1080/146608200300079536

2. Rascovsky K, Hodges JR, Knopman D, et al. Sensitivity of revised diagnostic criteria for the behavioural variant of frontotemporal dementia. *Brain*. Sep 2011;134(Pt 9):2456-77. doi:10.1093/brain/awr179

3. Strong MJ, Abrahams S, Goldstein LH, et al. Amyotrophic lateral sclerosis - frontotemporal spectrum disorder (ALS-FTSD): Revised diagnostic criteria. *Amyotrophic lateral sclerosis & frontotemporal degeneration*. 2017;18(3-4):153–174. doi:10.1080/21678421.2016.1267768

4. Kasper E, Schuster C, Machts J, et al. Dysexecutive functioning in ALS patients and its clinical implications. *Amyotroph Lateral Scler Frontotemporal Degener*. Jun 2015;16(3-4):160-71. doi:doi.org/10.3109/21678421.2015.1026267

5. Abrahams S, Leigh PN, Harvey A, Vythelingum GN, Grisé D, Goldstein LH. Verbal fluency and executive dysfunction in amyotrophic lateral sclerosis (ALS). *Neuropsychologia*. 2000;38(6):734-747. doi:10.1016/s0028-3932(99)00146-3

6. Grace J, Malloy PH. *Frontal Systems Behavior Scale (FrSBe): Professional Manual*. Psychological Assessment Resources (PAR); 2001.

7. Cedarbaum JM, Stambler N, Malta E, et al. The ALSFRS-R: a revised ALS functional rating scale that incorporates assessments of respiratory function. *J Neurol Sci*. 1999;169(1-2):13-21. doi:10.1016/s0022-510x(99)00210-5

8. Ashburner J. A fast diffeomorphic image registration algorithm. *NeuroImage*. 2007;38(1):95–113. doi:10.1016/j.neuroimage.2007.07.007

9. American College of Radiology. Phantom Test Guidance for Use of the Large MRI Phantom for the ACR MRI Accreditation Program. 2018.

10. Temp AGM, Naumann M, Hermann A, Glass H. Applied Bayesian Approaches for Research in Motor Neuron Disease. *Front Neurol*. 2022;13:796777. doi:10.3389/fneur.2022.796777
